# A Markerless Method for Genome Engineering in *Zymomonas mobilis* ZM4

**DOI:** 10.3389/fmicb.2019.02216

**Published:** 2019-10-11

**Authors:** Piyush Behari Lal, Fritz M. Wells, Yucai Lyu, Indro N. Ghosh, Robert Landick, Patricia J. Kiley

**Affiliations:** ^1^DOE Great Lakes Bioenergy Research Center, University of Wisconsin–Madison, Madison, WI, United States; ^2^Department of Biomolecular Chemistry, University of Wisconsin–Madison, Madison, WI, United States; ^3^College of Biological and Pharmaceutical Sciences, China Three Gorges University, Yichang, China; ^4^Department of Biochemistry, University of Wisconsin–Madison, Madison, WI, United States; ^5^Cell and Molecular Biology Graduate Training Program, University of Wisconsin-Madison, Madison, WI, United States; ^6^Department of Bacteriology, University of Wisconsin-Madison, Madison, WI, United States

**Keywords:** genome engineering of a non-model bacterium, green fluorescent protein, fluorescence activated cell sorting, recombineering suicide plasmid, biofuels, *Zymomonas mobilis*

## Abstract

Metabolic engineering of the biofuel-producing *Zymomonas mobilis* is necessary if we are to unlock the metabolic potential present in this non-model microbe. Manipulation of such organisms can be challenging because of the limited genetic tools for iterative genome modification. Here, we have developed an efficient method for generating markerless genomic deletions or additions in *Z. mobilis*. This is a two-step process that involves homologous recombination of an engineered suicide plasmid bearing *Z. mobilis* targeting sequences and a subsequent recombination event that leads to loss of the suicide plasmid and a genome modification. A key feature of this strategy is that GFP expressed from the suicide plasmid allows easy identification of cells that have lost the plasmid by using a fluorescence activated cell sorter. Using this method, we demonstrated deletion of the gene encoding lactate dehydrogenase (*ldh*) and the operon for cellulose synthase (*bcsABC*). In addition, by modifying the plasmid design, we demonstrated targeted insertion of the *crtIBE* operon encoding a neurosporene biosynthetic pathway into the *Z. mobilis* genome without addition of any antibiotic resistance genes. We propose this approach will provide an efficient and flexible platform for improved genetic engineering of *Z. mobilis*.

## Introduction

While the pace of research into genetic tools for model organisms has accelerated over the last half century, the development of tools for non-model bacteria has lagged behind. *Zymomonas mobilis* ZM4, an obligatory fermentative alpha-proteobacterium, is one such organism that lacks convenient tools for genetic engineering. This organism has garnered significant interest as a platform for the biosynthesis of biofuels because of its native ability to efficiently metabolize glucose to ethanol rather than to biomass (He et al., 2014; Yang et al., 2016; Wang et al., 2018). To optimize *Z. mobilis*’ metabolism and unlock its full potential for industrial fermentation of compounds of interest, methods for quickly and efficiently removing undesirable genes and pathways and adding novel genes and pathways are needed.

Existing genetic tools for *Z. mobilis* are not optimal for creation of strains with multiple engineered loci. A common approach to insertionally inactivate genes relies on host homologous recombination of an antibiotic resistance gene flanked by *Z. mobilis* DNA contained on a suicide plasmid, which, once integrated into the genome, can be screened for events leading to loss of delivery plasmid and gene of interest (Senthilkumar et al., 2004; Strazdina et al., 2018). This approach was enhanced by use of lambda red recombinase to increase the frequency of recombination events (Senthilkumar et al., 2004; Bo et al., 2013). However, the inability to remove the antibiotic resistance cassette quickly limits the number of modifications that can be made to a particular strain especially given the natural resistance of *Z. mobilis* to several antibiotics (Bochner et al., 2010). To overcome this problem, FLP recombinase was used to remove the antibiotic resistance gene to create a markerless gene deletion (Zou et al., 2012). However, introduction and removal of the stable plasmid bearing the FLP recombinase is a time-consuming process. To avoid the problems associated with using antibiotic resistance genes, Xia et al. (2018) made use of a sucrose counter-selection (*sacB*) to accelerate the process of integrated plasmid loss (Xia et al., 2018), but *Z. mobilis* ZM4 is annotated to also have *sacB*, questioning the general utility of this approach. Lastly, a CRISPR/Cas9 system was deployed to remove a native plasmid from *Z. mobilis* (Cao et al., 2017). This technique has not yet been extended to genome modification. Therefore, we sought to develop a flexible and an efficient method for genome engineering that would overcome some of the limitations of existing methods.

In this paper, we describe the creation of an efficient, reproducible, and flexible method for making markerless mutations in *Z. mobilis* by homologous recombination. We demonstrate its primary utility by making knockouts of the *ldh* gene (ZMO0256: lactate dehydrogenase) and the *bcsABC* genes (ZMO1083-85; cellulose synthase). We also describe how this technique could be extended to make markerless knock-ins at a desired locus by introducing carotenoid biosynthetic genes, *crtIBE*, from *Rhodobacter sphaeroides* (Takaichi and Maoka, 2015; Su et al., 2018). Herein, we also describe the suicide vector, which forms the basis for this technique. We also describe our unique choice to include the gene encoding GFP in our vector, which, through the use of a transilluminator or fluorescence activated cell sorting (FACS), allows us to track and screen for the presence or absence of the plasmid during intermediate steps in the process. This system should allow the facile and targeted addition or deletion of genes of interest in an iterative manner and thus facilitate the engineering of *Z. mobilis* for desirable traits.

## Materials and Methods

Primers, bacterial strains, and plasmids used in this study are listed in Tables 1–3.

**TABLE 1:** Primer list.

**Primer**	**Primer sequence (5′-3′)**
**number**	
P1	GTGCCGAGGATGACGATG
P2	CGTCATCCTCGGCACGATGACGATTGTGCCTTTG
P3	CAGAAAGCCTAATAACCCATAGCCTTACG
P4	CGTAAGGCTATGGGTTATTAGGCTTTCTG
P5	GCTACGCCTGAATAAGTGACCAGCAACTTTATCAGAC
P6	ACTTATTCAGGCGTAGCAC
P7	GACCAGCAACTTTATCAGAC
P8	GTCTGATAAAGTTGCTGGTCAGCGCTCGGTCTTGCCTT
P9	GGTGCTACGCCTGAATAAGTGCGCTTTTCCGCTGCATAAC
P10	GCGCTTTTCCGCTGCATA
P11	GTTATGCAGCGGAAAAGCGCATAGGCGTATCACGAGGC
P12	GGTGCTACGCCTGAATAAGTGTGCTTAAAACGCAAAAAGC
P15	ACCGAGCGCTACTAGTGATAAGCTGTCAAACATG
P16	TATCACTAGTAGCGCTCGGTCTTGCCTT
P17	AGCTTAACTAACTAGTTATATCGACCTTTTATTTTCAAAAATTTAATC
P18	GGTCGATATAACTAGTTAGTTAAGCTGCTATAAATATTATATTTTAC
P19	GCAAGGCAAGACCGAGCGCTTCAAGACCAGCAACTTTATC
P20	AGCGCTCGGTCTTGCCTT
P21	CGTTCGGCTGTTCTGGGTG
P22	AAACCCTGTAAGCCGGGAAGC
P23	TGGGCGTTTCCACTGTTGCG
P24	ATGTATATCTCCTTCTTAAAGTTAAAC
P25	TTTAAGAAGGAGATATACATATGCCCTCGATCTCGCCC
P26	TATTTATAATAGAAAGTAAAGACTAGATCGGGTTGGCCCG
P27	CCCGATCTAGTCTTTACTTTCTATTATAAATAAAGGAGACC TTTCATGAGGCACAAGATGGCGTTTGAACAGC
P28	GCAGGAATTCGATATCAAGCTCAGACGCGGGCCGCGAC
P29	GCTTGATATCGAATTCCTG
P30	ATACGCCTATTATATCGACCTTTTATTTTCAAAAATTTAATC
P31	GGTCGATATAATAGGCGTATCACGAGGCCCTTTCG
P32	TATAGCAGCTTAATCAGACGCGGGCCGCGAC
P33	CCGCGTCTGATTAAGCTGCTATAAATATTATATTTTACAAAATAATGC
P34	CGCTTGACCTTTACTTCGCC
P35	CAGGCTCACCCGCTAAAGTC
P36	CGAAATACCAGACGAACAGCCC
P37	ATCTATGCGGGTATCGGCGAC
P38	TCAACACAGGCTTCAACGAC

**TABLE 2:** Strain list.

**Strain**	**Description**	**Source or references**
WM6026	*E. coli lacIq rrnB*3 Δ*lacZ*4787 *hsdR*514 Δ*araBAD*567 Δ*rhaBAD*568 *rph*-1 *attλ*::pAE12(Δ*ori*R6K-*cat*:: Frt5), Δ*endA*::Frt *uidA*(ΔMluI)::pir *att*HK::pJK1006(Δ*ori*R6K-*cat*::Frt5; *trfA*::Frt) Δ*dapA*::Frt	Blodgett et al., 2007
DH5α	*E. coli* F^–^ *endA1 glnV44 thi*-*1 recA1 relA1 gyrA96 deoR nupG purB20* φ80d*lacZ*ΔM15 Δ(*lacZYA-argF*) U169, *hsdR*17(r_K_^–^m_K_^+^), λ^–^	Lab collection
*Z. mobilis*	*Zymomonas mobilis* ZM4 ATCC 31281	GLBRC
PK15556	*Z. mobilis ldh*_UP_::pPK15552	This work
PK15557	*Z. mobilis ldh*_UP_::pPK15535	This work
PK15569	*Z. mobilis ldh*_UP_::pPK15564	This work
PK15575	*Z. mobilis*Δ*ldh*::*crtIEB*	This work
PK15589	*Z. mobilis bcsABC*_UP_::pPK15538	This work
PK15597	*Z. mobilis*Δ*bcsABC*	This work
PK15598	*Z. mobilis*Δ*ldh*	This work

**TABLE 3:** Plasmid list.

**Plasmid**	**Description**	**References or source**
pRL814	Broad host range plasmid	Ghosh et al., 2019
pACYC184	*cat* p15A *ori tet*	Lab collection
pPK15252	Gibson assembly of p15A *ori* and *cat* (2115 bp of pACYC184 amplified by primers P_1_ and P_6_), 500 bp flanking regions of *Z. mobilis ldh* (*ldh*_UP_ and ldh_DN_ fragments)	This work
pPK15296	pPK15252 with *mob from* pSUP202 inserted between *cat* and *ldh*_DN_	This work
pPK15303	pPK15296 with RBS element and *gfp* gene from pRL814 inserted between *cat* and *mob*	This work
pPK15445	pPK15303 with optimized RBS for *gfp*	This work
pPK15534	pPK15445 with deletion of terminal 100 bp of p15A fragment to remove *tet* promoter and adjacent *ldh*_UP_ and *ldh*_DN_ replaced by a *Spe*I site	This work
pPK15535	pPK15445 with deletion of terminal 100 bp of p15A fragment to remove *tet* promoter and insertion of *Spe*I site between *ldh*_UP_ and *ldh*_DN_	This work
pPK15538	pPK15534 with 500 bp regions upstream and downstream of the *bcsABC* operon inserted at *Spe*I site	This work
pPK15564	pPK15535 with *crtIEB* cassette inserted at *Spe*I site between *ldh*_UP_ and *ldh*_DN_	This work
pPK15552	pPK15303 with deletion of terminal 100 bp of p15A fragment to remove *tet* promoter	This work
pSUP202	pMB1 replicon, Ap^R^, Tc^R^, Cm^R^, *mob*	Simon et al., 1983

### Growth of Bacterial Strains

*Zymomonas mobilis* strains were cultured in rich medium ZRMG (1% yeast extract, 0.2% KH_2_PO_4_, 2% glucose) or glucose-minimal medium (Martien et al., 2019) at 30°C. *Escherichia coli* strains were grown in Luria-Bertani (LB) medium. Chloramphenicol was used at a final concentration of 120 μg ml^–1^ for *Z. mobilis* and 20 μg ml^–1^ for *E. coli* strains. Spectinomycin was used at a final concentration of 100 μg ml^–1^ for *Z. mobilis* and 50 μg ml^–1^ for *E. coli* strains.

### Conjugation

*Escherichia coli* donor strain WM6026 (Blodgett et al., 2007) was used to conjugate plasmids into the recipient strain *Z. mobilis* ZM4. WM6026, a *m*-diaminopimelate (DAP) auxotrophic strain, was grown at 30°C aerobically in LB containing DAP (0.1 mM) with appropriate antibiotic. Overnight cultures were then subcultured into the same medium but lacking antibiotic to an OD_600_ of 0.15 and were grown to an OD_600_ of 0.5. *Z. mobilis* was incubated in 5.0 ml of ZRMG without shaking at 30°C until late exponential phase was reached. For conjugation, 1.0 ml of cells, adjusted to OD_600_ of 0.5, of both recipient and donor was mixed in a 2.0 ml microfuge tube. The cell suspension was briefly centrifuged at 17,300 × *g* for 30 s, and the supernatant was decanted. The pellet was resuspended in the small amount of remaining medium and placed as a drop on a prewarmed (30–37°C) ZRMG containing 1% tryptone, 0.15 mM DAP agar plate. Plates were incubated overnight at 30°C (12–15 h). Cells were collected from plates by resuspending into 1.0 ml ZRMG liquid media. The conjugation mixture was then vortexed for 10 s, spun at 17,300 × *g*, and the pellet was resuspended in 1.0 ml ZRMG liquid medium. The suspensions were incubated without shaking at 30°C for 2 h, and 100 μl of the undiluted, 10-fold, and 100-fold diluted cell suspension was plated on antibiotic-containing ZRMG agar plates. Plates were incubated at 30°C.

### Enrichment of Non-fluorescent Cells Using FACS

A single fluorescent exconjugant colony was grown in liquid ZRMG medium at 30°C without shaking. Twenty microliters of cell suspension from early log phase was diluted in 1 ml sterile 137 mM NaCl, 2.7 mM KCl, 10 mM Na_2_HPO_4_, 1.8 mM KH_2_PO_4_, pH 7.4, and loaded into a Sony MA900 or SH800 FACS equipped with a 70 μm sorting chip according to the manufacturer’s recommendations. Sorting of non-fluorescent events away from the major population of fluorescent cells was done using a 488 nm laser for excitation and 525/50 nm detector to measure emission. Sorted events were collected in 1 ml ZRMG liquid medium, a fraction of which was plated on ZRMG agar plates. Alternatively, one can screen for *gfp*^–^ cells by plating the recombinant cell suspension, grown without selection, on antibiotic-free rich medium plates to obtain ∼10,000 colonies and visually screening colonies that have lost fluorescence with a blue light transilluminator.

### PCR Amplification of DNA

For PCR amplification of DNA fragments to be used for cloning or sequence verification, the high fidelity Q5 polymerase from New England Biolabs was used. For screening or confirming genome modifications, GOTaq^*R*^ Flexi DNA polymerase (Promega) was used. For colony PCR, a single colony was picked and resuspended in 50.0 μl of distilled water. 2.0 μl of the suspension was used as a template for PCR amplification. Cell lysis was achieved by heating the PCR reaction mixture at 98°C for 30 s in a thermocycler before amplification steps. Annealing temperature and elongation time were followed as directed by the manufacturer’s specifications. Standard techniques were used for analysis or purification of DNA fragments or other molecular biology methods.

### Construction of Suicide Plasmid (pPK15534)

A conjugatable suicide plasmid (pPK15534) was constructed in several steps by PCR amplification of indicated DNA fragments followed by Gibson assembly using NEBuilder^®^ HiFi DNA Assembly Cloning Kit. DNA fragments containing the p15A origin of replication (*ori*) and a chloramphenicol resistance gene (*cat*) were amplified from pACYC184 (Chang and Cohen, 1978), and 500 bp regions upstream (*ldh*_UP_) and downstream (*ldh*_DN_) of the *ldh* coding region were amplified from the *Z. mobilis* genome, using primer pairs P1-P6, P2-P3, and P4-P5, respectively, to generate pPK15252. A plasmid mobilization element, *mob*, was amplified from pSUP202 (Simon et al., 1983) using primers P8-P9 and cloned at the junction of *ldh*_DN_ and *cat* in pPK15252, amplified by primers P7-P6 to generate pPK15296. A DNA fragment encoding the superfolder fluorescent protein, GFP, driven by the T7A1 promoter and a ribosome binding site was amplified from pRL814 (Ghosh et al., 2019) using primers P11-P12 and added to pPK15296, amplified by primers P6-P10 to generate pPK15303. The ribosome binding site was subsequently optimized using Salis’ algorithm (Salis et al., 2009; Borujeni et al., 2014) to improve GFP fluorescence in *Z. mobilis* by PCR amplification of pPK15303 using primers P13 and P14, which contain optimized RBS sequence in overhang regions to generate pPK15445. An orphan *tet* promoter was also removed and *Spe*I cloning site was introduced by Gibson assembly of two fragments of pPK15445 generated by a pair of primers P19-P18 and P17-P20, to generate pPK15535. Finally, to create a vector for cloning any target DNA (pPK15534), the orphan *tet* promoter, *ldh*_UP_ and *ldh*_DN_, were removed and replaced by a *Spe*I cloning site by Gibson assembly of a fragment of pPK15445 generated by a pair of primers P15-P16.

### Measurement of the Carotenoid Neurosporene

To measure the production of neurosporene, we followed an established protocol (Wraight et al., 1978). Briefly, cells were grown in ZRMG media to an OD_600_ of 0.5, the cell pellet from 2 ml culture was resuspended in 100 μl water, mixed with 1 ml of 7:2 mixture of acetone:methanol solvent, and spun for 3 min at 21,000 × *g*. The absorbance between 300 and 550 nm of the resulting supernatant was recorded on a Perkin Elmer Lambda 25 spectrophotometer.

## Results

### Overview

To create a more flexible system for deleting or adding genes iteratively to *Z. mobilis*, we rationalized that a recombination-based method was needed that did not require permanent introduction of antibiotic resistance genes into the genome (i.e., markerless modifications) but still could be rapidly screened for desired events. Creation of a conjugatable suicide vector containing GFP (pPK15534) allowed a more convenient method for tracking the loss of the recombineering suicide plasmid from strains, resulting in markerless gene deletions or insertions. Here, we describe the development and function of this system with three examples.

### Demonstration of an Efficient Conjugation System for Introducing DNA Into *Z. mobili*s

**FIGURE 1:**
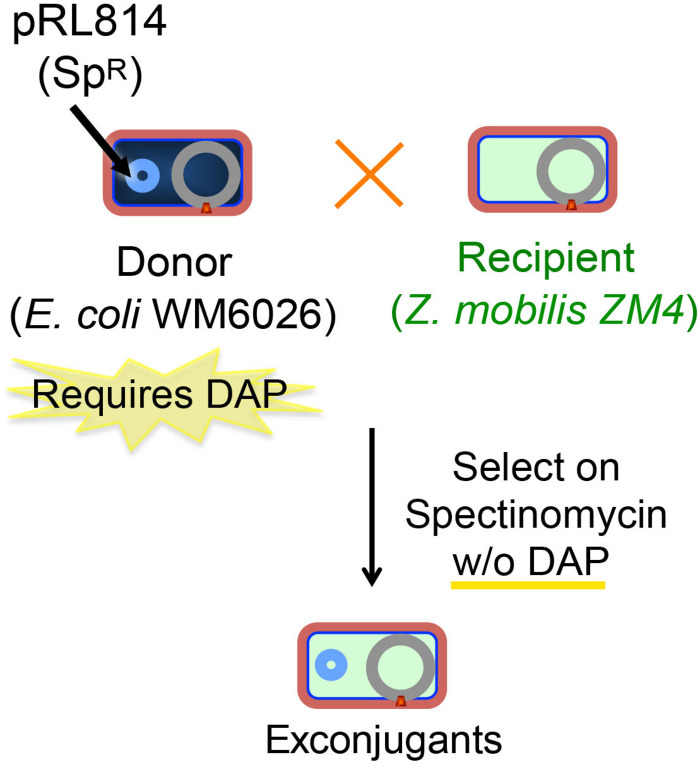
Scheme for conjugation of plasmids from *E. coli* to *Z. mobilis. Z. mobilis* (light blue) was conjugated using the DAP auxotrophic *E. coli* strain WM6026 (dark blue) as described in the section “Materials and Methods.” Briefly, donor and recipient strains were mixed and incubated in ZRMG containing tryptone and DAP overnight at 30°C. The conjugation mixture was recovered in liquid ZRMG medium at 30°C for 2 h, and plated in ZRMG with appropriate antibiotic.

To implement a gene-modification system our first step was to establish a reproducible and efficient conjugation method for introducing plasmids into *Z. mobilis* from *E. coli*. We took advantage of an existing *E. coli* donor strain that possesses a chromosomally encoded copy of the RP4 conjugation machinery able to transfer plasmids containing a mobilization element (*mob*) and is auxotrophic for DAP (Blodgett et al., 2007). When this *E. coli* donor strain containing the broad host range plasmid pRL814 was mated with *Z. mobilis* for an optimum period of 12–14 h at 30°C on a solid medium (Figure 1), we found a reproducible transfer frequency of 0.1% per input recipient cells; conjugations performed in liquid medium were generally unsuccessful, which was expected since conjugation by RP4 (IncP-type) machinery is enhanced on solid medium (Bradley et al., 1980). Importantly, this conjugation efficiency was robust enough to support the use of a suicide-plasmid homologous-recombination-based integration method; exconjugants should appear at a 100–1000-fold less frequency than that obtained with a stable plasmid to account for the additional recombination event.

### Designing a Mobilizable Suicide Plasmid pPK15534 for *Z. mobili*s

**FIGURE 2:**
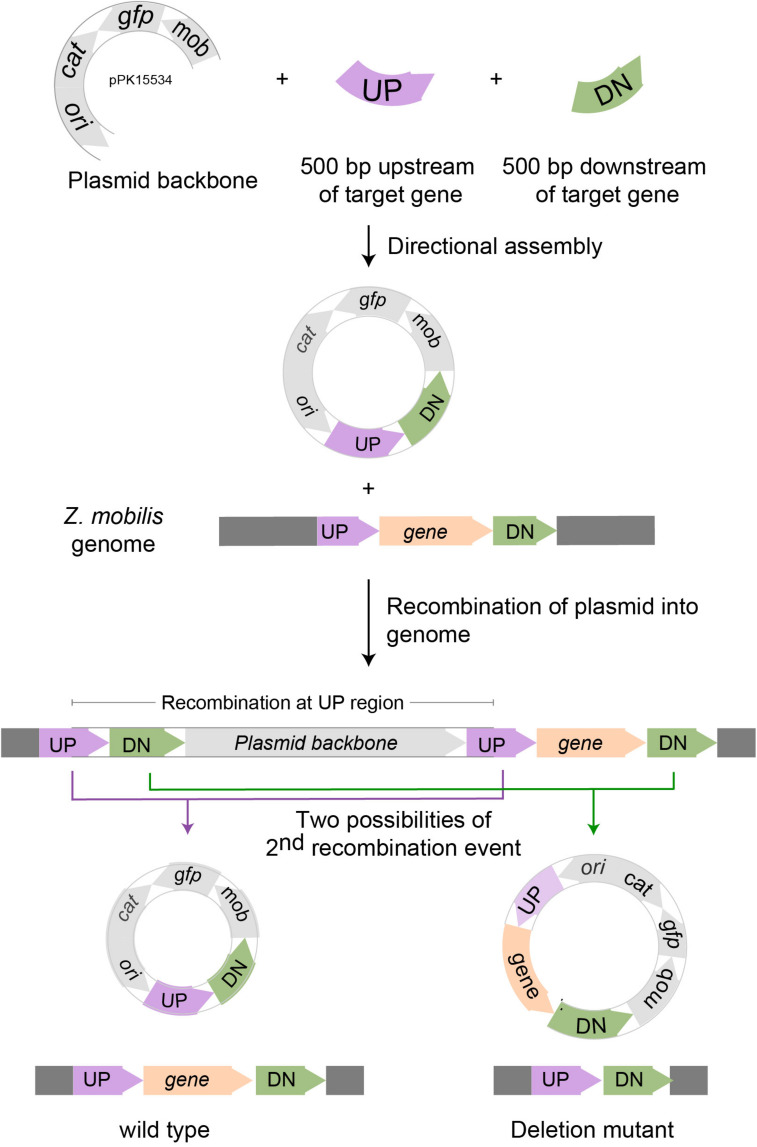
Scheme for deleting genes in *Z. mobilis*. Deletion of a gene in *Z. mobilis* is accomplished by cloning 500 bp of DNA from upstream (UP) of and downstream (DN) of the gene to be deleted into the suicide plasmid pPK15534. Next, the plasmid is introduced into *Z. mobilis* by conjugation and single crossover homologous recombination events are selected for with chloramphenicol. Shown here is a crossover that occurred at the UP location, but recombination at the DN location is equally possible. Lastly, growing cells without selection allow detection of a second recombination event that results in plasmid loss by screening for the loss of fluorescence from GFP. Wild-type and deletion mutant genotypes were distinguished by PCR amplification.

We designed the suicide plasmid to easily track genomic recombination events (Figure 2). The origin of replication, *oriC*, was selected from pACYC184, which was known not to replicate in *Z. mobilis.* The RP4 *mob* element from pSUP202 was chosen to enable conjugation from an *E. coli* donor strain (Simon et al., 1983). A single *Spe*I restriction site was introduced to facilitate cloning of target DNA to direct recombination of the plasmid into the *Z. mobilis* genome. Inclusion of *cat* on the plasmid backbone allowed selection of chloramphenicol resistance to identify single crossover recombination events in *Z. mobilis*. The superfolder *gfp* (Pedelacq et al., 2006), also on the plasmid backbone, allowed direct tracking of the plasmid and sorting by FACS. The latter enabled rapid enrichment of non-fluorescent cells that had undergone additional recombination events and lost the plasmid.

### pPK15534 Can Be Used as a Vehicle to Efficiently Delete Genes in *Z. mobili*s

**FIGURE 3:**
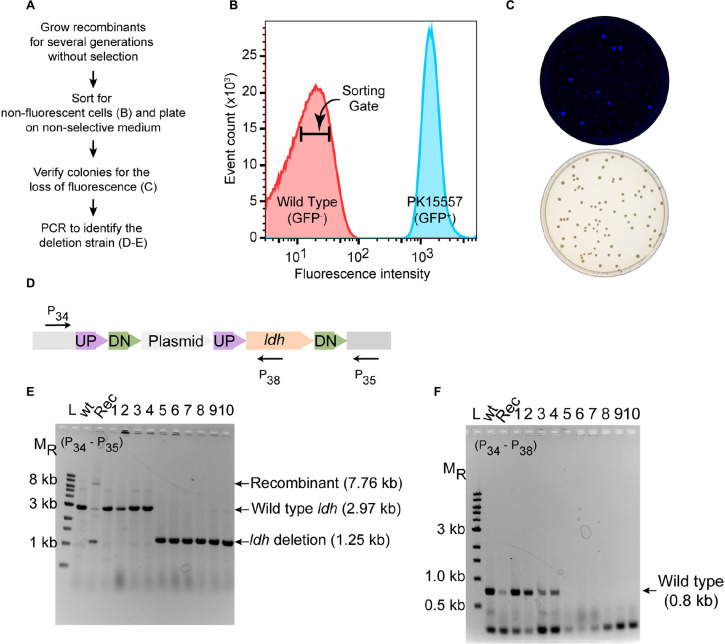
Deletion of *ldh* gene. **(A)** Workflow for enrichment of non-fluorescent cells and screening for the modified genome. **(B)** Plot from FACS of *Z. mobilis* strain PK15557 that has suicide plasmid (pPK15535) recombined into the genome. The *X*-axis represents fluorescence intensity and *Y*-axis represents the total number of sorted events. The sorting gate for collecting the non-fluorescent cells (red peak) is indicated by the bar. **(C)** Top plate shows the fluorescence image of colonies from FACS sorted non-fluorescent cells plated on non-selective media. Bottom plate shows the same colonies viewed with visible light. **(D)** The location of primers used to screen non-fluorescent candidates for wild-type (wt) and deletion genotypes by PCR is indicated by arrows. **(E,F)** Agarose gel electrophoresis of products from PCR amplification of 10 non-fluorescent-candidate colonies. Selected size markers are indicated on the left (M_*R*_). **(E)** Amplification using primers P34 and P35 yielded a 1.25-kb DNA fragment for the *ldh* deletion whereas the wt *ldh* DNA fragment was 2.97 kb. Strains that still possess the plasmid integrated into the genome (Rec) are expected to have a band of 7.8 kb. **(F)** Amplification using P34 and P38 yielded a DNA fragment of 0.8 kb, confirming those strains that still possess the *ldh* gene, while strains in which *ldh* has been deleted (six total) lacked the equivalent amplified fragment. Labels are the same as in panel **E**. Similar results were obtained when this experiment was biologically replicated three times.

As a proof of principle, we tested whether we could efficiently delete an average size gene, choosing the 1.7 kb *ldh* gene encoding lactate dehydrogenase (*ldh*, ZMO0256), since its function should be dispensable in *Z. mobilis* (Zou et al., 2012; Strazdina et al., 2018; Jones-Burrage et al., 2019). Our suicide plasmid (pPK15534) was engineered to contain two 500 bp sequences, immediately flanking but not including the target gene (pPK15535) (Figure 2). These sequences should direct homologous recombination of the plasmid into the genome, crossing over either upstream or downstream of the target gene within the genome. Following conjugation of the plasmid into *Z. mobilis*, we isolated Cm^*R*^ exconjugants at a frequency of 10^–5^, a reasonable frequency for a single recombination event following conjugation. We confirmed the integration location of one representative recombinant (PK15557) by PCR amplification across *ldh* using a pair of primers annealing at a region further upstream and downstream of the plasmid *ldh* targeting sequences (Figure 3D). The recombinant strain (shown in Figure 3E) also showed minor bands for both the genotype of wild type and the deletion of *ldh* indicating that these variants were present in the population at a low level.

To identify the rare cells in the population that underwent an additional crossover to lose the integrated plasmid, we took advantage of the fact that these cells will also be non-fluorescent because of the loss of *gfp.* We grew the recombinant strain for seven cell doublings without chloramphenicol selection and then enriched for the desired population of *gfp*^–^ cells by using a fluorescence-activated cell sorter (FACS). Cells were sorted to obtain 10,000 events that were binned into the gate indicative of non-fluorescent cells (Figures 3A,B). We typically observed that viability of the sorted cells was consistently 20% or less but only those sorted into the *gfp*^–^ bin. Possibly, in the nested gate that we use for *gfp*^–^ cells, unwanted dead cells or debris of size similar to the *Z. mobilis* cells also appear. Nevertheless, we routinely obtain hundreds of colonies after plating the non-fluorescent cells. Visualization of the plates with an imaging system showed that >90% of the colonies were non-fluorescent (Figure 3C).

To distinguish crossover events that regenerate wild type *ldh* from the *ldh* deletion, we used PCR amplification (Figures 3D–F). Of 10 non-fluorescent colonies screened by PCR, 6 isolates had a DNA fragment of the size expected for the *ldh* deletion strain (Figures 3D–F), whereas four were wild type. We showed that the deletion endpoints were as predicted from our plasmid design by Sanger sequencing of the *ldh* gene region (Supplementary Figure S1). Thus, because the FACS sorting step takes just a few minutes, this strategy for enriching for non-fluorescent cells and then screening non-fluorescent colonies by PCR amplification allowed rapid identification of strains containing the deletion of interest with no other markers.

### Deletion of *bcsABC* Operon

**FIGURE 4:**
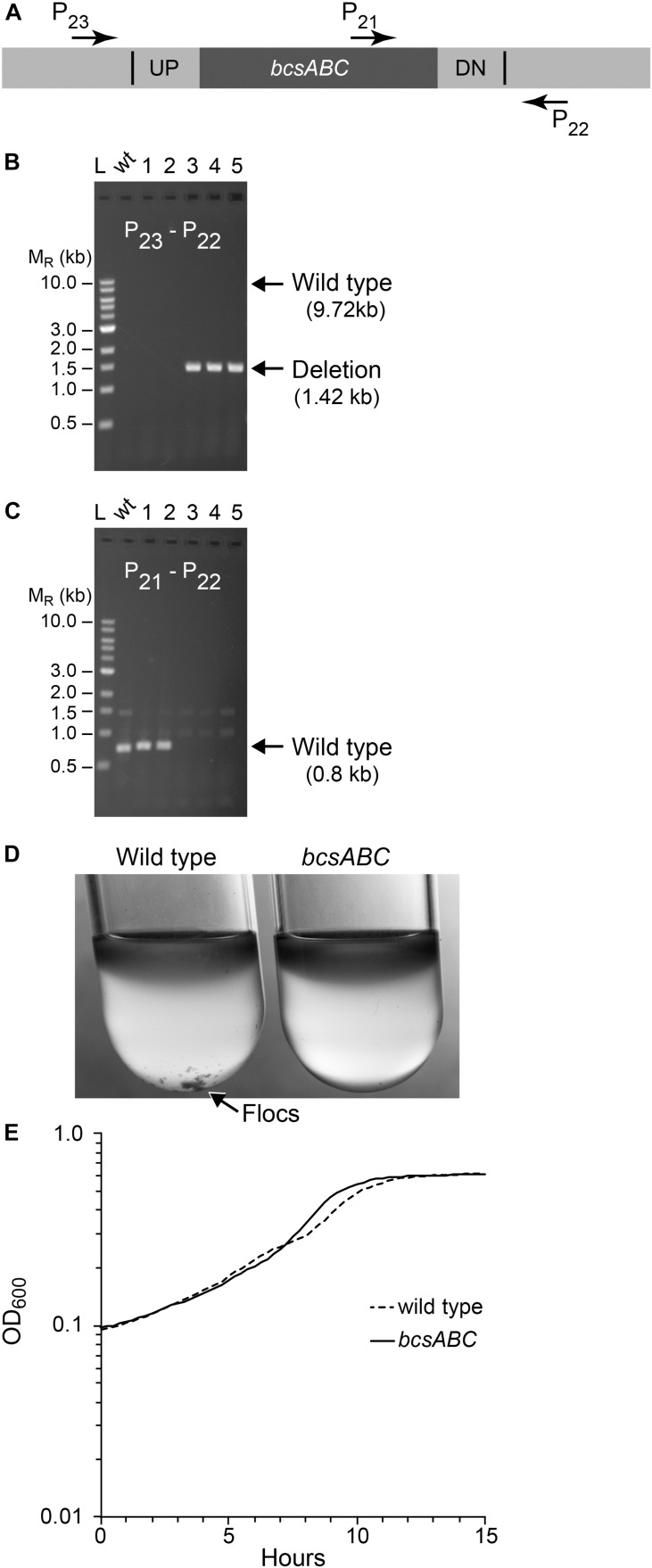
Deletion of *bcsABC* operon. Non-fluorescent deletion candidates for *bcsABC* were isolated using the same workflow as outlined for *ldh* and screened for deletions or wild-type (wt) alleles by PCR amplification. **(A)** The location of primers used to distinguish between the wt and the deletion genotype is indicated by arrows. **(B,C)** Agarose gel electrophoresis of products from PCR amplification of five non-fluorescent candidate colonies. **(B)** Amplification using primers P_22_ and P_23_ yielded a DNA fragment of 1.4 kb for the deletion of *bcsABC* (three total), while wt cells are expected to produce an amplicon of ∼10 kb, which was poorly amplified under our reaction *conditions. **(C)** To verify the genotype of wt strains, a second PCR experiment using primers P21 and P22 was included. In this case, wt strains are expected to yield an amplicon of 0.7 kb while the deletion strain should not produce a band. Similar results were obtained from six biological replicates of this workflow. **(D)** To test the phenotype of our mutant, we compared flocculation of the wt (left) and deletion strain (right) after growth for 15 h in glucose minimal media under aerobic conditions. Flocs are indicated by the arrow. **(E)** Growth curve of wt and mutant strains grown in glucose minimal media under anaerobic conditions.*

Next, we tested whether we could efficiently delete a larger size operon, choosing the 8.3 kb *bcsABC* operon, encoding functions for cellulose synthesis, since its function is known to be dispensable in *Z. mobilis* (Deutschbauer et al., 2014; Xia et al., 2018; Jones-Burrage et al., 2019). In addition, elimination of *Z. mobilis bcsABC* should minimize flocculation in liquid medium, which hinders accurate measurements of optical density (OD_600_) (Figure 4). Our suicide plasmid was engineered to contain two 500 bp sequences, immediately flanking the *bcsABC* operon (pPK15538). The plasmid was introduced by conjugation, and recombinants were confirmed by PCR. Following culturing without selection, we sorted cells to obtain 10,000 non-fluorescent events and plated for single colonies. Of five non-fluorescent colony candidates screened by PCR, three were confirmed to have the desired deletion of the *bcsABC* operon (Figure 4). We showed that the deletion endpoints were as predicted from our plasmid design by Sanger sequencing of the *bcs* gene region (Supplementary Figure S1). This example shows that deletion of a longer sequence does not affect the efficiency of obtaining deletions. To determine if the strain lost the flocculating phenotype, we grew the wild type and the deletion strain in minimal medium without shaking. As expected, wild type produced flocs while the deletion strain did not produce flocs (Figure 4D). Further, growth curves with the deletion strain showed a smooth curve unlike wild type (Figure 4E).

### Insertion of *crtIEB* Genes Into the Genome

**FIGURE 5:**
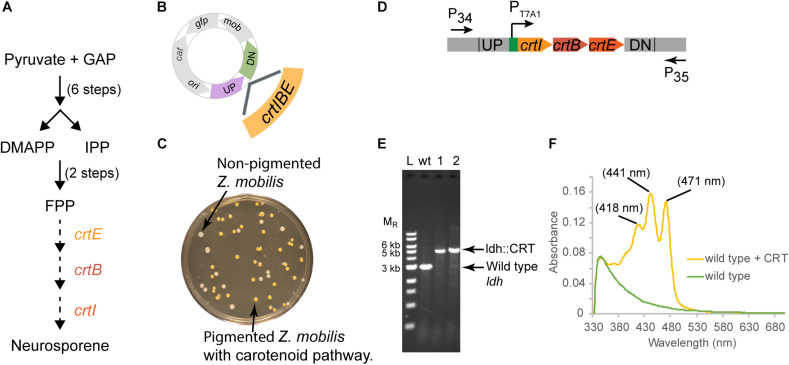
Insertion of a functional CRT cassette. **(A)** A heterologous pathway for production of the carotenoid neuroporene by introduction of the *crtIBE* genes from *Rhodobacter sphaeroides.* Dotted arrows indicate the reactions mediated by the *R. sphaeroides* gene products. **(B)** Schematic of suicide plasmid used to deliver *crtIBE* to *Z. mobilis ldh.* UP and DN are the same 500 bp sequences used to delete the *ldh* gene. **(C)** Non-fluorescent candidates containing *crtIBE* replacing *Z. mobilis ldh* were isolated using the same workflow as outlined for *ldh* and screened for insertions first by the presence of orange colored colonies. **(D)** The location of primers used to distinguish between the wt and the insertion genotype is indicated by arrows. **(E)** Agarose gel electrophoresis of products from PCR amplification of orange colored colonies. Amplification using primers P_34_ and P_35_ yielded a DNA fragment of 5.4 kb for the insertion of *crtIBE*, whereas wt cells are expected to produce an amplicon of 2.9 kb. Selected size markers are indicated on the left (M_*R*_). **(F)** Absorption spectrum of acetone:methanol extracts from orange colored colonies containing *crtIBE* compared to wt. Peak maxima characteristic of neurosporene are marked. We examined more than five isolates for insertion of the CRT cassette in independent experiments, and measured neurosporene from three different isolates. We conducted two independent experiments both resulting in the successful insertion of the CRT cassette at the *ldh* locus.

For strain engineering, a critical tool is to be able to equip bacteria with novel pathways to produce products of interest. Thus, we also tested whether we could use our suicide plasmid to deliver heterologous genes and recombine them into the genome. An advantage of this method is that these added genes would be stably maintained on the genome and would require no added antibiotics for selection. We demonstrate this by replacing the *ldh* gene with the heterologous *crtIEB* operon (CRT) which produces an easily detected orange-pigmented carotenoid, neurosporene (Figure 5A) from *R. sphaeroides* (Su et al., 2018).

In this case, we placed the *crtIEB* genes from *R. sphaeroides* between the *ldh* targeting sequences on plasmid pPK15564 (Figure 5B). We also optimized RBSs (Salis et al., 2009; Borujeni et al., 2014) upstream of each gene. The plasmid was introduced by conjugation, and recombinants were confirmed by PCR. Following culturing without selection, we sorted cells to obtain 10,000 non-fluorescent events and plated for single colonies. Visualization of the plates indicated that some of the non-fluorescent colonies were also distinctly orange (Figure 5C). PCR screening of two non-fluorescent pigmented colonies confirmed the insertion of the operon at the *ldh* gene site (Figures 5D,E). We showed that the insertion junctions were as predicted from our plasmid design by Sanger sequencing (Supplementary Figure S1). Spectral analysis of acetone–methanol extracts of these strains showed an absorbance spectrum characteristic of neurosporene (Figure 5F; Takaichi and Maoka, 2015).

### Optimization of Expression of Genes in *Z. mobili*s

**FIGURE 6:**
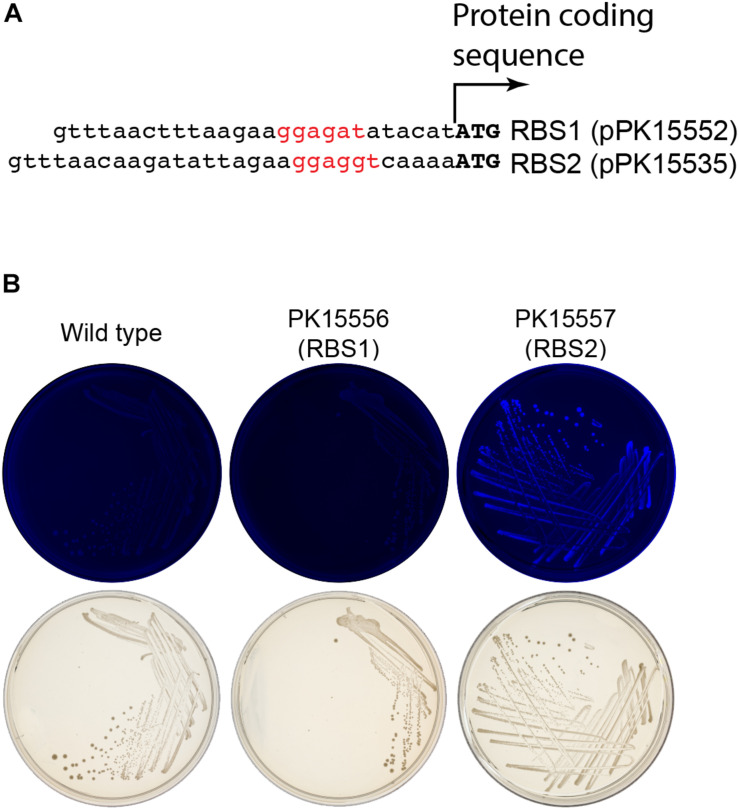
Enhancement of *gfp* expression to increase colony fluorescence. **(A)** Comparison of the ribosome binding sites (rbs) from pPK15535 plasmid containing the optimized *gfp* compared to the original suicide plasmid pPK15552, in which *gfp* possessed a ribosome binding site from pRL814. This latter variant failed to produce detectable fluorescence in *Z. mobilis* in single copy. The RBS was improved using the Salis program (Salis et al., 2009; Borujeni et al., 2014). **(B)** Top plates show the fluorescence images of colonies from indicated integrated plasmids strains excited at 490 nm and imaged at 510 nm. Bottom plates show the same colonies viewed with visible light. Only the cells possessing GFP translated from the improved RBS have detectable fluorescence.

We initially encountered insufficient expression of *gfp* in *Z. mobilis* for differentiating fluorescent from non-fluorescent colonies or cells. The ribosome binding site for *gfp*, originally obtained from a stable vector pRL814 was sufficient to produce fluorescent colonies from a plasmid but not when integrated into the genome (*Z. mobilis* PK15556, Figure 6B). To improve the robustness of the GFP fluorescence screening, the ribosome binding site of *gfp* was modified guided by the results from the RBS calculator for maximizing predicted RBS strength (Salis et al., 2009; Borujeni et al., 2014). The difference between the original sequences and the optimized ribosome-binding site that we used to optimize expression of *gfp* for our suicide plasmid is shown in Figure 6A. Similar optimization of the *crtIEB* genes using the RBS calculator was required to detect pigmented colonies for neurosporene production (data not shown).

## Discussion

This work describes the development of a new flexible method for genome engineering in *Z. mobilis*, and how this method can be used for both knocking out genes and introducing novel sequences. We demonstrated the general utility of our genome engineering method by creating two targeted gene deletion strains and by adding a functional heterologous operon to a defined position in the *Z. mobilis* genome. The suicide plasmid and general approach should also be useful to other non-model organisms that benefit from the simple broad host range conjugation delivery system and recombination-based strategy.

Currently, deletion of genes in *Z. mobilis* relies on recombineering strategies to replace a gene typically with an antibiotic resistance marker cassette. Removal of this marker to allow subsequent genome modification has been demonstrated by a recombination reaction using FLP recombinase (Zou et al., 2012) when the marker is flanked with FRT sequences. However, this approach is time consuming due to the additional steps needed to introduce and remove the stable plasmid encoding FLP recombinase. In addition, FRT sequences remain in the genome providing sites for unwanted recombination, which could negatively impact iterative strain design. Counter selection with *sacB* on sucrose media (Xia et al., 2018) is feasible but relies on the desired phenotype or the native *Z. mobilis sacB* from not negatively impacting the sucrose selection. Our approach of screening for the loss of GFP fluorescence should not negatively impact the desired phenotype as far as we are aware.

We also showed that the frequency of a single crossover event in *Z. mobilis* averages 0.3%, which indicates that growth of a recombinant strain for just a few cell doublings generates sufficient cell mass to screen for a second recombination event. FACS facilitated the easy isolation of these rare events from the total population by sorting. Overall, this approach allows creation of strains containing a markerless addition or removal of a gene in two recombination events *ad infinitum.* This approach will be extremely useful in introducing genes for new pathways into *Z. mobilis*, which can be added at any position in the genome. By simply altering the *Z. mobilis* sequences flanking the genes of interest on the suicide plasmid, genes can be added to replace native ones, can be incorporated into native operons, or as stand alone operons, demonstrating the versatility of this approach. Our method is also not limited to FACS availability, since one can screen for non-fluorescent colonies by plating the recombinant cell suspension grown without selection and visually identify colonies that have lost the fluorescence using an imager.

## Conclusion

In summary, our method has several highly desirable attributes; namely, it is markerless, it can be greatly accelerated with FACS, and it leverages the simple and efficient process of conjugation. Critical to this technique’s success, especially for use with FACS, was our optimization of the expression of *gfp* by RBS optimization. This enabled the visualization of fluorescent colonies and sorting of cells by FACS from a single, chromosomally inserted copy of *gfp*. We expect our method to be of value to genetic studies in the field, and our introduction of the use of FACS to enrich for desired phenotypes to be of exceptional value to *Z. mobilis* studies in the future. Finally, this method provides a simple approach for metabolic engineering where iterative strategies for genome engineering are necessary to build strains with desirable traits through sequence addition or deletion for producing molecules of interest.

## Data Availability Statement

All datasets generated for this study are included in the manuscript/Supplementary Files.

## Author Contributions

PL and PK conceived the overall study. PL, PK, RL, and IG designed the experiments. PL, FW, and YL carried out the experiments. PK and PL drafted the manuscript and all authors agreed on the final version of the manuscript.

## Conflict of Interest

The authors declare that the research was conducted in the absence of any commercial or financial relationships that could be construed as a potential conflict of interest.

## Supplementary Material

FIGURE S1 Sequence of the modified genome of *Z. mobilis* strains. To verify the structure of the insertion or deletion strains, we sequenced across the junction regions of genomic DNA. **(A)** For the *ldh* deletion strain, genomic DNA was amplified using primer P34 and P35 and sequenced using primer P34. **(B)** For deletion of *bcsABC*, genomic DNA was amplified using primer P22 and P23 and sequenced using primer P22. **(C,D)** For insertion of the CRT cassette at the *ldh* locus, we amplified upstream of the CRT cassette using primer P34 and P36 and sequenced using primer P34 **(C)** and amplified downstream of the CRT cassette using primer P37 and P35 **(D)** and sequenced using primer P35. Colored arrows indicate junctions of deletion or insertion of heterologous sequence. Top line represents expected sequence of the DNA fragment and the bottom line represents the sequencing data.
