# Corrigendum: A Markerless Method for Genome Engineering in *Zymomonas mobilis* ZM4

**DOI:** 10.3389/fmicb.2021.719621

**Published:** 2021-06-29

**Authors:** Piyush Behari Lal, Fritz M. Wells, Yucai Lyu, Indro N. Ghosh, Robert Landick, Patricia J. Kiley

**Affiliations:** ^1^DOE Great Lakes Bioenergy Research Center, University of Wisconsin–Madison, Madison, WI, United States; ^2^Department of Biomolecular Chemistry, University of Wisconsin–Madison, Madison, WI, United States; ^3^College of Biological and Pharmaceutical Sciences, China Three Gorges University, Yichang, China; ^4^Department of Biochemistry, University of Wisconsin–Madison, Madison, WI, United States; ^5^Cell and Molecular Biology Graduate Training Program, University of Wisconsin-Madison, Madison, WI, United States; ^6^Department of Bacteriology, University of Wisconsin-Madison, Madison, WI, United States

**Keywords:** genome engineering of a non-model bacterium, green fluorescent protein, fluorescence activated cell sorting, recombineering suicide plasmid, biofuels, *Zymomonas mobilis*

In the original article, there were two mistakes in Figure 3 as published. The X-axis label of Figure 3B should be 10^3^ rather than 10^2^. Figure 3D contained a word inversion in the text “UP-Plasmid-UP-DN-*ldh*-DN” and should read “UP-DN-Plasmid-UP-*ldh*-DN.” The corrected Figure 3 appears below.

**Figure 3:**
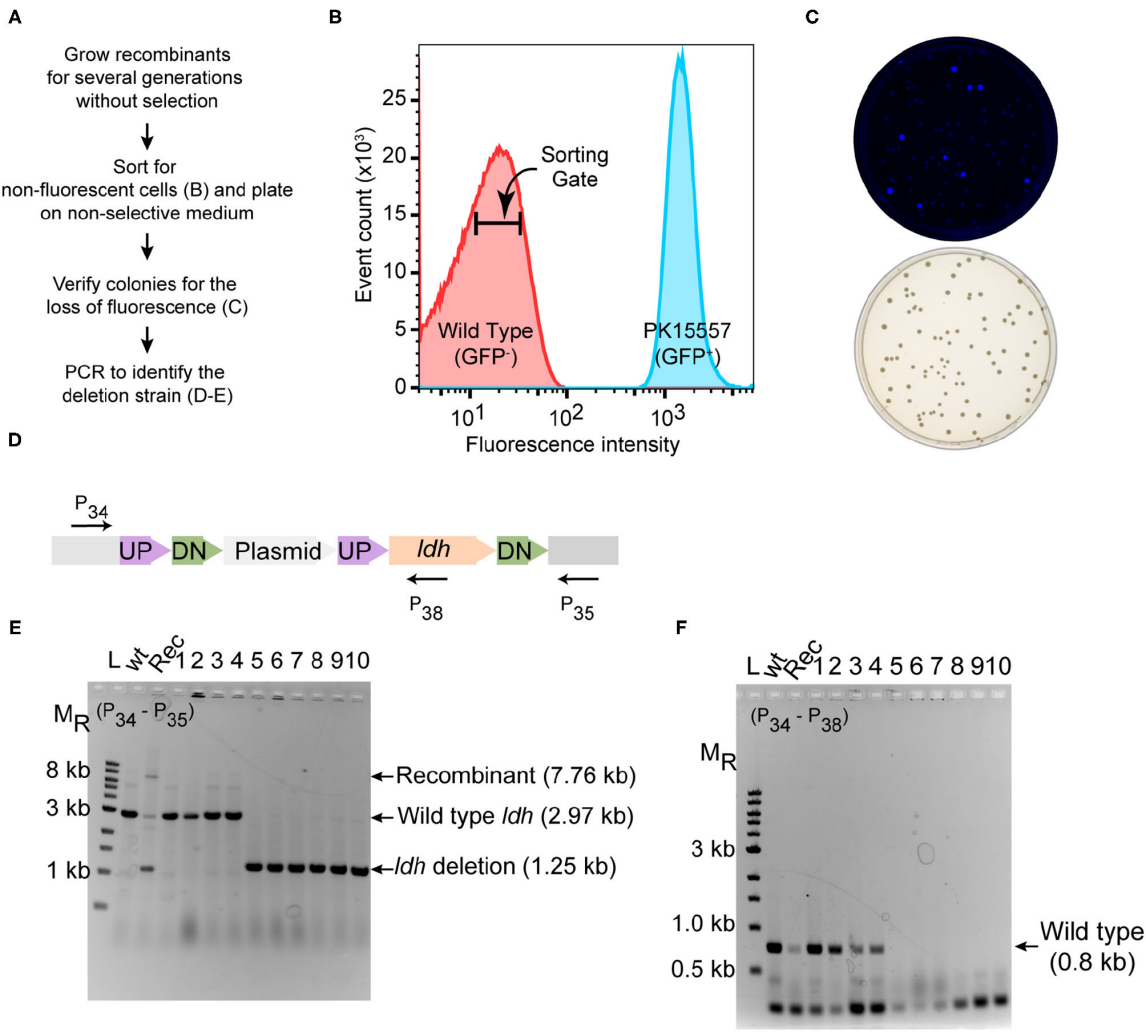
Deletion of ldh gene. **(A)** Workflow for enrichment of non-fluorescent cells and screening for the modified genome. **(B)** Plot from FACS of Z. mobilis strain PK15557 that has suicide plasmid (pPK15535) recombined into the genome. The X-axis represents fluorescence intensity and Y-axis represents the total number of sorted events. The sorting gate for collecting the non-fluorescent cells (red peak) is indicated by the bar. **(C)** Top plate shows the fluorescence image of colonies from FACS sorted non-fluorescent cells plated on non-selective media. Bottom plate shows the same colonies viewed with visible light. **(D)** The location of primers used to screen non-fluorescent candidates for wild-type (wt) and deletion genotypes by PCR is indicated by arrows. **(E,F)** Agarose gel electrophoresis of products from PCR amplification of 10 non-fluorescent-candidate colonies. Selected size markers are indicated on the left (MR). **(E)** Amplification using primers P34 and P35 yielded a 1.25-kb DNA fragment for the ldh deletion whereas the wt ldh DNA fragment was 2.97 kb. Strains that still possess the plasmid integrated into the genome (Rec) are expected to have a band of 7.8 kb. **(F)** Amplification using P34 and P38 yielded a DNA fragment of 0.8 kb, confirming those strains that still possess the ldh gene, while strains in which ldh has been deleted (six total) lacked the equivalent amplified fragment. Labels are the same as in panel E. Similar results were obtained when this experiment was biologically replicated three times.

The authors apologize for this error and state that this does not change or affect the scientific conclusions of the article in any way. The original article has been updated.

