# Supplementary figures and images for: A Markerless Method for Genome Engineering in Zymomonas mobilis ZM4
Source: Front Microbiol. 2019 Oct 11;10:2216. doi: 10.3389/fmicb.2019.02216 (PMC6797605; doi:10.3389/fmicb.2019.02216)

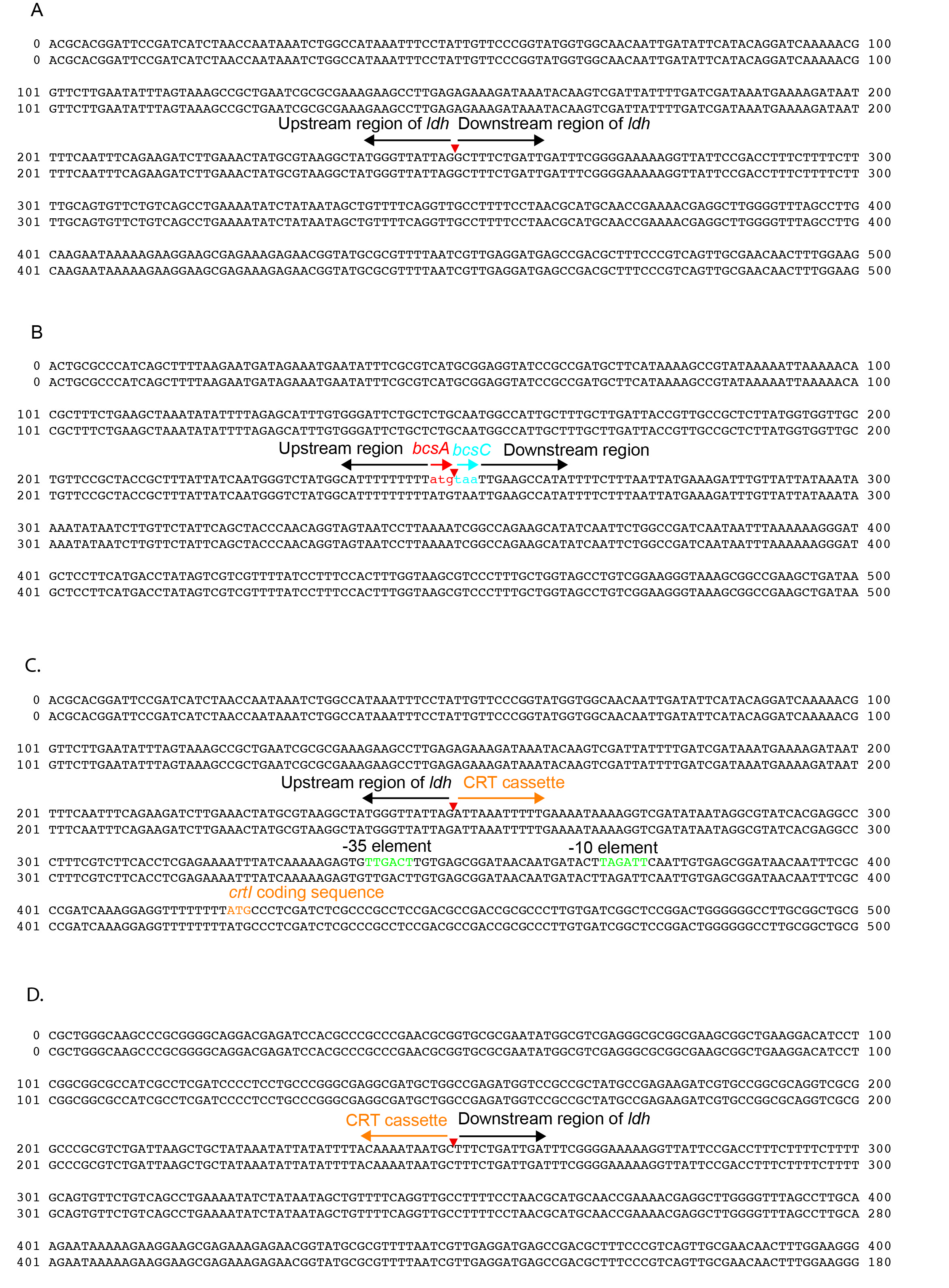

Supplement: FIGURE S1 — Sequence of the modified genome of Z. mobilis strains. To verify the structure of the insertion or deletion strains, we sequenced across the junction regions of genomic DNA. (A) For the ldh deletion strain, genomic DNA was amplified using primer P34 and P35 and sequenced using primer P34. (B) For deletion of bcsABC, genomic DNA was amplified using primer P22 and P23 and sequenced using primer P22. (C,D) For insertion of the CRT cassette at the ldh locus, we amplified upstream of the CRT cassette using primer P34 and P36 and sequenced using primer P34 (C) and amplified downstream of the CRT cassette using primer P37 and P35 (D) and sequenced using primer P35. Colored arrows indicate junctions of deletion or insertion of heterologous sequence. Top line represents expected sequence of the DNA fragment and the bottom line represents the sequencing data. [file Image_1.JPEG]
